# Antihypertensive Agents and Risk of Parkinson's Disease: A Nationwide Cohort Study

**DOI:** 10.1371/journal.pone.0098961

**Published:** 2014-06-09

**Authors:** Yen-Chieh Lee, Chin-Hsien Lin, Ruey-Meei Wu, Jou-Wei Lin, Chia-Hsuin Chang, Mei-Shu Lai

**Affiliations:** 1 Department of Family Medicine, Cathay General Hospital, Taipei, Taiwan; 2 Department of Neurology, National Taiwan University Hospital, Taipei, Taiwan; 3 Department of Medicine, College of Medicine, National Taiwan University, Taipei, Taiwan; 4 Cardiovascular Center, National Taiwan University Hospital Yun-Lin Branch, Dou-Liou City, Yun-Lin County, Taiwan; 5 Institute of Preventive Medicine, College of Public Health, National Taiwan University, Taipei, Taiwan; Osaka University Graduate School of Medicine, Japan

## Abstract

**Background and Purpose:**

Hypertension has been associated with Parkinson's disease (PD), but data on antihypertensive drugs and PD are inconclusive. We aim to evaluate antihypertensive drugs for an association with PD in hypertensive patients.

**Methods:**

Hypertensive patients who were free of PD, dementia and stroke were recruited from 2005–2006 using Taiwan National Health Insurance Database. We examined the association between the use of calcium channel blockers (CCBs), angiotensin converting enzyme inhibitors (ACEIs), angiotensin receptor blockers (ARBs) and the incidence of PD using beta-blockers as the reference. Cox regression model with time-varying medication use was applied.

**Results:**

Among 65,001 hypertensive patients with a mean follow-up period of 4.6 years, use of dihydropyridine CCBs, but not non-dihydropyridine CCBs, was associated with a reduced risk of PD (adjusted hazard ratio [aHR] = 0.71; 95% CI, 0.57–0.90). Additionally, use of central-acting CCBs, rather than peripheral-acting ones, was associated with a decreased risk of PD (aHR = .69 [55–0.87]. Further decreased association was observed for higher cumulative doses of felodipine (aHR = 0.54 [0.36–0.80]) and amlodipine (aHR = 0.60 [0.45–0.79]). There was no association between the use of ACEIs (aHR = 0.80 [0.64–1.00]) or ARBs (aHR = 0.86 [0.69–1.08]) with PD. A potentially decreased association was only found for higher cumulative use of ACEIs (HR = 0.52 [0.34–0.80]) and ARBs (HR = 0.52 [0.33–0.80]).

**Conclusions:**

Our study suggests centrally-acting dihydropyridine CCB use and high cumulative doses of ACEIs and ARBs may associate with a decreased incidence of PD in hypertensive patients. Further long-term follow-up studies are needed to confirm the potential beneficial effects of antihypertensive agents in PD.

## Introduction

Parkinson's disease (PD) is a common neurodegenerative disorder which underlying mechanism leading to dopaminergic neuron death remains elusive and current therapies remain purely symptomatic [1]–[4]. Recent population-based cohort studies suggest that PD is associated with several cardiovascular risk factors, such as diabetes mellitus and hypertension [5], [6]. Data from one population-based cohort study of Finland shows that, as compared with normotensive subjects, women with hypertension are associated with a 60% increased risk of PD [6]. Therefore, the role of antihypertensive drugs in risk of PD is worth to be explored.

Increasing evidence has suggested that L-type calcium channels and the central renin-angiotensin system play a role in PD [7]–[9]. The age-dependent reliance on L-type calcium channel in dopaminergic neurons contributes to increased intracellular oxidative stress [7]. Angiotensin II, the effector peptide of the central renin-angiotensin system (RAS) in substantia nigra, is a pro-inflammatory compound that can activate the oxidative cascades with resulting neuronal death [10]. These *in vitro* studies form the bases of hypothesis that antihypertensive agents, especially angiotensin receptor blockers (ARBs), inhibitors of angiotensin converting enzyme (ACEIs), and calcium channel blockers (CCBs), may have possible neuroprotective effects in PD [11]–[15].

Few epidemiologic studies have examined the association between antihypertensive agents use and PD with inconsistent results [11], [17]–[21]. One recently published cohort study demonstrated that use of one subclass of CCBs that targets L-type calcium channels is associated with decreased PD incidence and mortality [18]._ENREF_11 The possible reasons that studies comparing the risk of PD between CCB users and non-users have different results may come from the age of the study participants, definition of drug exposure, and criteria for PD diagnosis. Furthermore, the effect of other classes of antihypertensive drugs on the development of PD is largely unknown.

Given that hypertension per se is a possible risk factor for PD [6], the comparison of antihypertensive drugs users with nonusers is susceptible to confounding by indication. We therefore restricted the enrolment to patients with hypertension receiving antihypertensive treatment to increase the homogeneity of our study cohort. We aim to examine the effects of different classes of antihypertensive agents on the risk of PD as compared to beta-blockers in hypertensive patients in a population-based cohort. Beta-blockers, especially Atenolol, were chosen as the reference because they are one of the commonly used drugs for the treatment of hypertension in Taiwan and have poor ability to cross the blood-brain-barrier [22].

Due to Taiwan's National Health Insurance Reimbursement Policy request, treatment of hypertension followed the American Heart Association guidelines; that is the target blood pressure depends on patients' risk level. For patients with low (<10%) or moderate (10–20%) 10-year Framingham risk, the target blood pressure is <140/90 mmHg. For patients with high Framingham risk (≥20%), such as patients with diabetes, chronic kidney disease, previous history of stroke, or established heart failure, the target blood pressure is <130/80 mmHg [23], [24].

## Methods

### Data Source

The enrollment rate in the single-payer, compulsory National Health Insurance program in Taiwan was 99%. The National Health Insurance Research Database (NHIRD) stores national data from demographic and enrollment records, hospital claims, ambulatory care visits, and pharmacy dispensing claims from hospitals, outpatient clinics, and community pharmacies. The Longitudinal Health Insurance Database 2005 comprises a random sample of one million subjects from the NHIRD.

### Study Population

We enrolled patients with hypertension during the recruitment period of January 1, 2005–December 31, 2006. Patients were included if they had at least one hospital admission with a diagnostic code of hypertension (ICD9-CM code 401) or two or more outpatient visits with a hypertension diagnostic code. The date of the first recorded code was defined as the index date. Patients were excluded if they 1) already had a diagnosis of PD, dementia, or cerebrovascular disease, 2) aged less than 50 years, or 3) did not have continuous insurance coverage during the 12 months before the index date. The study was approved by the National Taiwan University Hospital Research Ethics Committee.

### Use of Study Drugs

Four classes of commonly used antihypertensive medications were studied and identified via anatomical therapeutic chemical [ATC] codes: 1) beta-blockers (BBs); 2) calcium channel blockers (CCBs); 3) angiotensin converting enzyme inhibitors (ACEIs); and 4) angiotensin receptor blockers (ARBs). Patients with hypertension may have received combination anti-hypertensive drug therapy or switched from one class of drug to another. We assumed that the patients' exposure to each class of drugs independently and continuously contributed to their long-term PD risk. We also calculated the cumulative use of each class of medication and assigned the level of use to one of four categories based on the defined daily dose (DDD), which is the assumed average maintenance dose per day.

### Diagnosis of PD

The outcome of interest was defined as an outpatient visit or hospitalization involving a diagnosis of PD made by a neurologist (ICD-9-CM code 332). In a validation study examining 1985 PD patients in the National Taiwan University Hospital, a movement disorder specialist (CHL) evaluated the medical records of these patients and found a 94.8% of diagnostic accuracy.

### Covariate Ascertainment and Adjustment

We used inpatient and outpatient diagnosis and prescription files during the 12-month period before the index date to ascertain the patients' medical histories and medication use (ICD-9-CM codes and ATC codes are provided in Table S1 in File S2).

### Statistical Analysis

The crude incidence rates of PD and their 95% confidence intervals (CIs) were estimated based on a Poisson distribution. A Cox regression model with time-varying use of antihypertensive agents was used to calculate the hazard ratios (HR) using BBs as the reference group. We also examined potential dose-response relationships of antihypertensive use on the incidence of PD.

Because the number of PD cases was small in comparison to the number of covariates reflecting participants' baseline characteristics, we included disease risk score quintiles as summary measures of all these covariates to deal with baseline imbalance among different anti-hypertensive users. Using a logistic regression model, we estimated the disease risk score using indicators for antihypertensive use by group, age, sex, underlying diseases, and concomitant medications during the 12 months before the index date [22]. Additionally, time-varying use of medications including statins, anti-diabetics, anti-platelets, NSAIDs, and anti-psychotics after the index date was also adjusted in the outcome model.

We conducted sensitivity analyses using three different definitions of PD: first, including subjects with any inpatient/outpatient diagnosis of PD as well as those receiving anti-Parkinson drugs (levodopa, bromocriptine mesylate, pergolide mesylate, amantadine, selegiline, cabergoline, ropinirole, or pramipexole); second, excluding subjects with PD occurring within one year after cohort entry. Given that PD may have a prodromal period, we therefore conducted a third sensitivity analysis that included a lag-time by excluding all participants' antihypertensive drugs exposure 1.5 years before the diagnosis of PD to avoid protopathic bias.

Participants were further stratified for subgroup analysis according to 1) age (<65, ≥65 years), and 2) sex. Analysis of possible interactions was performed using the likelihood ratio test. Two-sided *p* values <0.05 were considered to be statistically significant. All statistical analyses were performed using SAS 9.2 (SAS Institute, Cary, NC).

## Results

**Figure 1:**
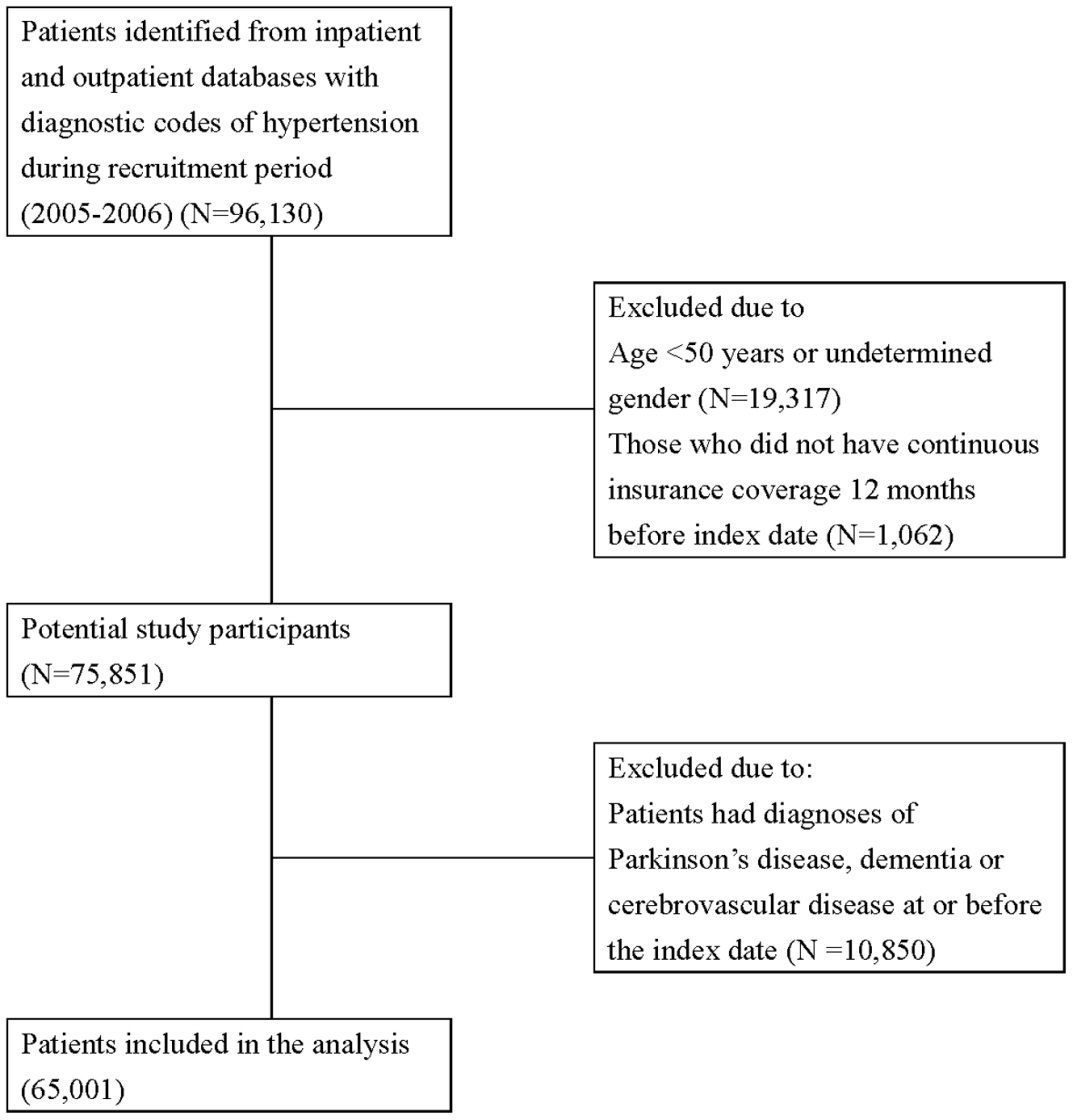
Flowchart of study cohort assembly from the National Taiwan Insurance Database.

A total of 65,001 patients with hypertension were included in the analysis (Figure 1). Several baseline characteristics and medications were associated with an increased risk of PD (Table 1). The number of patients in each category of antihypertensive drug use and combination therapies was shown in table S2 in File S1. The average follow-up duration was 4.6 years. The crude incidence rate for PD was 8.09 per 1,000,000 person-days for the overall hypertensive population, and 8.50, 7.85, 7.81, and 9.56 per 1,000,000 person-days for BBs, CCBs, ACEIs, and ARBs use, respectively. Compared with BBs, the use of CCBs was associated with a significantly reduced risk of PD (adjusted HR, 0.75; 95% CI, 0.59–0.96). This decreased association between CCBs and PD was most obviously observed in those with highest cumulative dosages (Table 2). Similar results with a trend of decreased risk were found in the sensitivity analyses using different outcome definitions, although risk estimates became non-significant due to smaller numbers of events occurring (Table 2). In addition, although there was no association between PD and the use of ACEIs or ARBs, a decreased association was observed in those with highest cumulative dosages of ACEIs (adjusted HR, 0.52; 95% CI 0.34–0.80) and ARBs (adjusted HR 0.52; 95% CI, 0.33–0.80) in the dose-response analysis (Table 2). The results remained similar after stratifying the hypertensive population according to age (≧ 65 or <65years) and gender (Table S3 in File S1). The potential protective effect of a higher cumulative dose of CCBs was only significant in women and elderly patients. The highest cumulative doses of ACEIs and ARBs were also associated with a significantly lower risk of PD in women and elderly patients (table S3 in File S1).

**Table 1:** Demographic data, comorbidities, medication use, and resource utilization in the study population and risk factors associated with Parkinson's disease.

	Total population (N = 65,001)	Odds ratio (95% CI)
*Patient characteristics*		
Age at diagnosis, mean (SD)	65.9 (9.94)	1.07 (1.06–1.08)
Male (%)	46.1	1.15 (0.99–1.34)
Index year of hypertension diagnosis (%)		0.91 (0.77–1.09)
2005	75.2	
2006	24.8	
*Cormorbidities (%)*		
Diabetes mellitus	25.2	1.43 (1.23–1.68)
Ischemic heart disease	15.0	1.65 (1.38–1.97)
Myocardial infarction	0.79	1.99 (1.09–3.64)
Angina	5.88	1.29 (0.97–1.71)
Heart failure	3.64	1.21 (0.84–1.73)
Migraine	1.58	1.43 (0.87–2.36)
Gout	12.6	1.24 (1.01–1.53)
Peripheral vascular disease	1.57	1.54 (0.95–2.50)
Chronic liver disease	11.0	0.96 (0.76–1.22)
Chronic obstructive pulmonary disease	14.9	1.54 (1.29–1.85)
Chronic kidney disease	7.89	1.46 (1.15–1.85)
Seizure	0.55	1.82 (0.86–3.86)
Rheumatoid arthritis	3.36	1.18 (0.80–1.72)
Osteoarthritis	22.6	1.76 (1.51–2.06)
Osteoporosis	6.90	1.47 (1.15–1.89)
Depression	3.53	2.17 (1.63–2.89)
Anxiety disorder	12.7	1.77 (1.48–2.13)
Bipolar disorder	0.34	2.93 (1.37–6.24)
Psychotic disorder	0.60	2.89 (1.62–5.17)
Peptic ulcer disease	17.4	1.87 (1.58–2.20)
Thyroid disease	0.38	1.42 (0.86–2.35)
Cancer	4.39	1.16 (0.83–1.62)
*Medication use (%)*		
COX-2 non-selective NSAIDs	74.9	1.25 (1.05–1.50)
COX-2 selective NSAIDs	9.35	1.70 (1.38–2.10)
Anti-platelet agents	30.9	1.28 (1.10–1.50)
Warfarin	0.61	2.09 (1.08–4.06)
Statins	14.5	1.02 (0.83–1.26)
Nitrates	11.1	1.74 (1.43–2.11)
Anti–diabetic agents	21.7	1.44 (1.22–1.70)
Insulin	2.75	1.95 (1.40–2.72)
Fibrates	6.42	0.91 (0.67–1.25)
Diuretics	27.2	1.55 (1.33–1.81)
Anti-arrhythmic agents	2.02	2.08 (1.43–3.03)
Estrogen	3.55	0.66 (0.41–1.07)
Digoxin	2.56	1.63 (1.12–2.36)
Anti–psychotics	10.0	1.57 (1.28–1.94)
Anti-depressants	8.39	1.73 (1.40–2.15)
Anti-epileptics	5.39	2.38 (1.89–2.99)
Thyroid therapy	1.59	1.42 (0.86–2.35)
Anti-gout preparations	13.9	1.24 (1.02–1.52)
*Resource utilization (median ± SD)*		
Total number of outpatient visits	27 (22.4)	1.01 (1.01–1.02)
Mean cost per outpatient visit (NTD)	766 (1,531)	1.00 (1.00–1.00)
Number of different specialists visit	6 (2.79)	1.11 (1.08–1.14)
Total number of Hospitalization	0.23 (0.72)	1.11 (1.04–1.18)
Mean cost per hospitalization (NTD)	6,781 (26,199)	1.00 (1.00–1.00)
Mean days per hospitalization	1.22 (9.73)	1.00 (1.00–1.01)

COX, cyclooxygenase; NSAID, non-steroid anti-inflammatory drugs; SD, standard deviation

**Table 2:** Hazard ratios for Parkinson's disease associated with calcium channel blockers, angiotensin converting enzyme inhibitors, and angiotensin receptor blockers as compared with beta blockers in hypertension population.

	Calcium channel blockers		ACE inhibitors		Angiotensin receptor blockers	
	Crude HR	Adjusted HR^a^	Crude HR	Adjusted HR^a^	Crude HR	Adjusted HR^a^
Main analysis^b^	0.89 (0.70–1.14)	0.75 (0.59–0.96)	0.79 (0.63–1.00)	0.80 (0.64–1.00)	0.93 (0.72–1.16)	0.86 (0.69–1.08)
Sensitivity analysis^c^	0.92 (0.71–1.19)	0.78 (0.61–1.01)	0.77 (0.61–0.97)	0.79 (0.62–1.00)	0.90 (0.72–1.14)	0.87 (0.69–1.10)
Sensitivity analysis^d^	1.03 (0.78–1.38)	0.83 (0.62–1.10)	0.87 (0.67–1.13)	0.84 (0.65–1.09)	1.02 (0.79–1.31)	0.92 (0.71–1.19)
Sensitivity analysis^e^	1.03 (0.79–1.33)	0.86 (0.66–1.12)	0.96 (0.74–1.23)	0.92 (0.71–1.19)	0.89(0.69–1.15)	0.82 (0.63–1.02)
Dose effect^f^						
1^st^ (Lowest quartile)	0.73 (0.54–1.00)	0.79 (0.58–1.07)	1.12 (0.78–1.62)	1.11 (0.77–1.61)	0.98 (0.67–1.45)	1.00 (0.68–1.48)
2^nd^	0.85 (0.65–1.11)	0.92 (0.70–1.21)	0.70 (0.45–1.08)	0.73 (0.47–1.13)	0.75 (0.49–1.16)	0.78 (0.51–1.20)
3^rd^	0.51 (0.37–0.70)	0.53 (0.38–0.73)	0.75 (0.51–1.12)	0.82 (0.55–1.21)	0.77 (0.52–1.14)	0.78 (0.53–1.16)
4^th^ (Highest quartile)	0.66 (0.50–0.87)	0.61 (0.46–0.81)	0.49 (0.32–0.75)	0.52 (0.34–0.80)	0.54 (0.35–0.83)	0.52 (0.33–0.80)

a Stratified by baseline disease risk score deciles and adjusted for time-varying co-morbidities and medications use (statins, anti-diabetes, anti-platelets, non-steroidal anti-inflammatory drugs, anti-psychotics, ischemic heart disease, heart failure, chronic kidney disease, and chronic obstructive pulmonary disease).

b Parkinson's disease defined as outpatient visit diagnosis of Parkinson's disease made by neurologists or any hospitalization diagnosis of Parkinson's disease.

c Parkinson's disease defined as inpatient or outpatient diagnosis of Parkinson's disease as well as receiving anti-Parkinson drugs.

d Parkinson's disease defined as diagnosis made after one year of follow-up.

e Exposure defined by 1.5 years of lag time.

f Calculated as quartiles of cumulative defined daily dose (DDD) of the drug class category.

ACE, angiotensin converting enzyme; HR, hazard ratio.

While we divided CCBs into dihydropyridine and non-dihydropyridine agents, any use of dihydropyridine CCBs, rather than non-dihydropyridine, was associated with a decreased risk of PD (adjusted HR, 0.71; 95% CI, 0.57–0.90). A further decreased risk was found for higher cumulative use, suggesting a potential dose-response effect of dihydropyridine CCBs in PD (Table 3). Due to the differences in lipophilic properties, we further distinguished CCBs into the central-acting ones that could cross the blood-brain barrier (felodipine, nifedipine, lercanidipine, nitrendipine and lacidipine) and the peripheral-acting ones that are thought to not cross blood-brain barrier as readily (amlodipine, verapamil, and diltiazem). We found any use of central-acting CCBs, rather than peripheral-acting ones, was associated with a decreased risk of PD (adjusted HR, 0.69; 95% CI, 0.55–0.87, table S4). Among the individual dihydropyridine CCB, the use of felodipine was found to have a reduced association with PD, with a potential dose-response relationship (HR, 0.72; 95% CI, 0.54–0.95 for any use; HR, 0.54; 95% CI, 0.36–0.80 for higher cumulative use). There was also a decreased association between higher cumulative use of amlodipine and risk of PD (Table 4).

**Table 3:** Hazard ratios for Parkinson's disease associated with dihydropyridine and non-dihydropyridine calcium channel blockers, as compared with beta blockers in hypertension population.

	Dihydropyridine		Non-dihydropyridine	
	Crude HR	Adjusted HR^a^	Crude HR	Adjusted HR^a^
Main analysis	0.79 (0.63–0.99)	0.71 (0.57–0.90)	0.95 (0.67–1.34)	0.73 (0.52–1.04)
Dose effect^e^				
1^st^ (Lowest quartile)	0.69 (0.50–0.95)	0.73 (0.53–1.01)	0.87 (0.41–1.83)	0.71 (0.34–1.50)
2^nd^	0.83 (0.63–1.11)	0.93 (0.70–1.24)	1.02 (0.53–1.97)	0.87 (0.45–1.68)
3^rd^	0.58 (0.43–0.80)	0.62 (0.45–0.85)	0.88 (0.44–1.77)	0.72 (0.36–1.45)
4^th^ (Highest quartile)	0.61 (0.46–0.82)	0.57 (0.42–0.76)	0.71 (0.39–1.30)	0.57 (0.31–1.03)

a Stratified by baseline disease risk score deciles and adjusted for time varying co-morbidities and medications use (statins, anti-diabetes, anti-platelets, non-steroidal anti-inflammatory drugs, anti-psychotics, ischemic heart disease, heart failure, chronic kidney disease, and chronic obstructive pulmonary disease).

e Calculated as quartiles of cumulative defined daily dose (DDD) of the drug class category.

ACE, angiotensin converting enzyme; HR, hazard ratio.

**Table 4:** Hazard ratios for Parkinson's disease associated with individual dihydropyridine calcium channel blockers, as compared with beta blockers in hypertension population.

	Any use		Low dose^b^		High dose^b^	
	Crude HR	Adjusted HR^a^	Crude HR	Adjusted HR^a^	Crude HR	Adjusted HR^a^
Amlodipine	0.87 (0.70–1.08)	0.83 (0.67–1.03)	0.92 (0.71–1.19)	0.95 (0.73–1.23)	0.60 (0.45–0.79)	0.60 (0.45–0.79)
Felodipine	0.69 (0.52–0.91)	0.72 (0.54–0.95)	0.89 (0.62–1.27)	0.95 (0.66–1.36)	0.51 (0.34–0.77)	0.54 (0.36–0.80)
Nifedipine	0.93 (0.71–1.23)	0.80 (0.61–1.06)	0.75 (0.50–1.10)	0.70 (0.47–1.04)	0.85 (0.60–1.21)	0.77 (0.54–1.10)
Lercanidipine	0.70 (0.36–1.35)	0.63 (0.33–1.22)	0.84 (0.35–2.03)	0.77 (0.32–1.85)	0.55 (0.21–1.49)	0.50 (0.19–1.34)
Nitrendipine	0.53 (0.26–1.05)	0.57 (0.28–1.14)	0.76 (0.32–1.84)	0.82 (0.34–1.98)	0.34 (0.11–1.07)	0.37 (0.12–1.17)
Lacidipine	1.05 (0.43–2.52)	0.99 (0.41–2.38)	0.44 (0.06–3.11)	0.39 (0.05–2.75)	1.59 (0.59–4.24)	1.60 (0.60–4.29)

a Stratified by baseline disease risk score deciles and adjusted for time varying co-morbidities and medications use (statins, anti-diabetes, anti-platelets, non-steroidal anti-inflammatory drugs, anti-psychotics, ischemic heart disease, heart failure, chronic kidney disease, and chronic obstructive pulmonary disease).

b Calculated as quartiles of cumulative defined daily dose (DDD) of the drug class category.

HR, hazard ratio.

## Discussion

Our study showed that use of CCBs in hypertensive patients has a dose-dependent decreased association with PD compared to use of BBs. This potentially beneficial effect was most obvious with the use of central-acting dihydropyridine CCBs. Additionally, although there was no association between the use of ACEIs or ARBs with risk of PD, a potentially decreased association was found for high cumulative use of these two classes of antihypertensive agents.

Although *in vitro* evidence has suggested that the increased reliance on L-type Cav1.3-calcium channels in dopaminergic neurons with advanced age accelerate the degeneration process, studies related to use of CCBs on reducing the incidence of PD are inconsistent. Three studies comparing the risk of PD between CCBs users and non-users showed that current long term use of dihydropyridine CCBs, especially central-acting ones, was associated with a reduced risk of PD (summarized in Table 5). However, another study demonstrated that current, but not past, dihydropyridine CCBs use was associated with a reduced risk of PD, even including peripherally-acting amlodipine and non-dihydropyridine CCBs [25]. Our study, which compared the risk of PD between difference classes of antihypertensive drugs and non-brain penetrating beta-blockers, reinforced the role of centrally acting dihydropyridine CCBs, especially Felodipine, in decreasing the incidence of PD in hypertensive patients. The potential beneficial effects of central-acting dihydropyridine CCBs in PD may come from the unique property of this class of CCBs specifically target Cav1.3 L-type calcium channels and have a much higher concentration in the brain than peripheral acting ones [7]–[9]. Although the non-dihydropyridine CCBs verapamil and diltiazem can also transverse the blood-brain barrier, neither is known to bind to the Cav1.3 L-type Ca+2 channels.6 Further studies are needed to explore the potential disease-modifying effect of CCBs in PD disease course. These observations form a basis of studies to test whether central-acting dihydropyridine CCBs would slow down disease progression or even neuroprotection in susceptible subjects. However, one recent trial failed to show isradipine, a central-acting dihydropyridine CCB, in delaying parkinsonism progression [25]. In addition to felodipine, our results showed that among dihydropyrine CCB, a further decreased association was also observed for higher cumulative doses of amlodipine. Similar to our findings, one recent nationwide cohort study also found that the beneficial effects of dihydropyridine CCB on the risk of PD were most obvious for amlodipine and felodipine [16]. Given that amlodipine dose not cross blood-brain barrier as readily as felodipine [19], we believe that amlodipine may have some other effects, rather than antagonist effects on calcium channels in the central nervous system. Further studies are needed to confirm the disease-modifying effect of central-acting CCBs in PD disease course.

**Table 5:** Summary for published reports examining associations between anti-hypertensive agents with Parkinson's disease.

Reference	Study design	Country	Study population	Comparative group	Number of participants	Drug use lag time	Confounding factors	Results
Paganini-Hill et al., 2001 [23]	Retrospective, Case-control	US	NA	Users vs. non-users	395 PD patients and 2320 controls	No	Sex, birth date	Decreased risk of PD with blood pressure medication OR 0.63 (0.48–0.80).
Ton et al., 2007 [21]	Retrospective, Case-control	US	Aged 35–89	Users vs. non-users	206 new PD patients and 383 controls	5 years prior to the index date	Age, sex, smoking, duration of enrollment, clinic	OR was 0.85 (0.43–1.66) for ever use of CCB, 1.20 (0.71–2.03) for BB. No relation to dose/duration.
Becker et al., 2008 [15]	Retrospective, Case-control (GPRD)	UK	Aged ≧40	Users vs. non-users	3,637 new PD patients and 3,637 controls	No	Age, sex, GP, duration of enrollment, smoking, BMI, underline disease	OR for current use of ≧ 30 prescriptions was 0.77 (0.63–0.65) for CCB, 1.08 (0.85–1.37) for ACEI, 0.91 (0.41–2.00) for ARB, 1.16 (0.95–1.41) for BB. No association for past use of CCB.
Louis et al., 2009 [9]	Case-control and prospective cohort analysis	Spain	Aged ≧65	Users vs. non-users	81 prevalent PD cases and 4663 controls; 3942 participants for follow up	No	Age, sex, education, depressive symptoms	Antihypertensive medication use including CCB was not associated with prevalent or incident PD
Ritz et al., 2010 [19]	Retrospective, Case-control	Denmark	Aged ≧35	Users vs. non-users	1,931 new hospital PD patients and 9,651 controls	2 years prior to the index date	Age, sex, COPD, Charlson's index, other antihypertensive drugs	OR was 0.70 (0.52–0.94) for ever use of L-type, centrally acting dihydropyridine CCB without dose or duration-response. OR, 1.29 (1.13–1.48) for BB, 1.11 (0.93–1.32) for ACEI, 0.94 (0.74–1.19) for ARB.
Simon et al., 2010 [20]	Prospective cohort study (NHS and HPFS)	US	Aged 30–55 (NHS) and 40–75(HPFS)	Users vs. nonusers or never users of CCB	120,530 women form NHS and 50,825 men from HPFS	No	Age, smoking, hypertension, BMI, physical activity, caffeine, alcohol, and total energy intake	No association between PD and baseline use of CCB, or other antihypertensives.
Pasternak et al., 2012 [16]	Prospective cohort study	Denmark	Aged ≧45	New users vs. never users of CCB	2,573,281 persons	No	Age, sex, year, propensity score, and use of other antihypertensive drugs and statins	RR 0.71 (0.60–0.82) for current use of dihydropyridine CCB, 1.04 (0.87–1.24) for past use, and 0.64 (0.42–0.96) for non-dihydropyridine CCB.

NA, not available; US, United States; UK, United Kingdom; PD, Parkinson's disease; OR, odds ratio, RR, rate ratio; NHS, Nurses' Health Study; HPFS, Health Professionals Follow-up Study; BB, beta-blocker, CCB, calcium channel blocker, ARB, Angiotensin II receptor blockers. The parentheses represents 95% confidence interval.

In our study, we also observed that high cumulative doses of ACEIs and ARBs were also associated with a decreased incidence of PD compared to beta-blockers, which beneficial effects was not present at low cumulative doses. Previous animal studies have suggested that ACEIs and ARBs may be neuroprotective due to their antioxidant properties [13]–[15]. Supportively, one double-blind placebo-controlled trial have shown that four weeks of treatment of perindopril, an ACEI, produced an improvement in the motor response to levodopa among patients with moderately severe PD [13]. Our results further support the notion that the dopaminergic systems interact with RAS in nigral-basal ganglia circuits [10].

The strength of our study was its relatively homogeneous population and the comparison of PD risk between different classes of antihypertensive medications. This nationally representative cohort involved a large sample size. Data collection from the NHI pharmacy database rather than self-reported questionnaires reduced the misclassification of exposure. Furthermore, covariates including underlying diseases, especially diabetes mellitus [26], medication use, and healthcare utilization prior to initiation of antihypertensives were taken into consideration. We made adjustments for the use of medications potentially affecting PD risk, such as NSAIDs [27], anti-diabetic agents [28], statins [29], [30], and neuroleptic agents.

However, our study has several limitations. First, the information regarding the blood pressure levels, which have been shown to be associated with risk of PD [6], was not available in the claims dataset. Further longitudinal study including serial measures of blood pressures over time is needed to clarify the interrelated roles of blood pressure level, use of anti-hypertensive agents, and PD. Second, vascular parkinsonism is a potential co-morbidity in patients with hypertension. Nonetheless, we tried to validate our diagnosis by excluding participants who had cerebrovascular diseases before PD diagnosis to avoid including vascular parkinsonism. We also attempted to validate our findings by using more stringent diagnostic criteria in the sensitivity analysis. Third, we could not exclude the possibility that some hypertensive patients already had subclinical or early PD at study entry. However, misclassification of the non-differential outcome in all antihypertensive agent groups would bias the study results toward the null hypothesis. In addition, the results were similar when we excluded patients who were diagnosed with PD within one year of study entry. In addition, smoking is known to decrease risk of PD. We were unable to directly control for smoking due to lack of data. Instead, we adjusted for COPD, since it may serve as a proxy for heavy smoking. Other confounding factors, such as the consumption of tea or coffee, other lifestyle-related factors and parameters of vascular function, such as brachial-ankle pulse wave velocity and extent of carotid artery atherosclerosis were not included in the study. Finally, PD patients with hand tremor could possibly be misdiagnosed with essential tremor and treated with beta-blockers. However, it is unlikely that the observed association between antihypertensives and PD was due to the use of beta-blockers as a reference group, as the reverse association was observed for dihydropyridine but not non-dihydropyridine CCBs in our population.

## Conclusions

We observed that use of centrally-acting dihydropyridine CCBs and higher doses of ACEIs and ARBs had a decreased association with PD compared to that of beta-blockers in hypertensive patients. Further long-term follow up studies are needed to confirm the potential use of antihypertensive agents in PD management.

## Supporting Information

Checklist S1 **STROBE checklist in the current study.**
(DOCX)

File S1 **Tables S1–S4.** Table S1: ICD-9-CM codes and ATC codes used in this study. Table S2: The number of patients in each category of antihypertensive medications and combination therapies. Table S3: Hazard ratios for Parkinson's disease associated with calcium channel blockers, angiotensin converting enzyme inhibitors and angiotensin receptor blockers as compared with beta blockers among different subgroups of patients with hypertension. Table S4: Hazard ratios for Parkinson's disease associated with central and peripheral-acting calcium channel blockers, as compared with beta blockers in patients with hypertension.
(DOC)
