# Supplementary material for: Antihypertensive Agents and Risk of Parkinson's Disease: A Nationwide Cohort Study
Source: PLoS One. 2014 Jun 9;9(6):e98961. doi: 10.1371/journal.pone.0098961 (PMC4049613; doi:10.1371/journal.pone.0098961)
Supplement: File S1 — Tables S1–S4. Table S1: ICD-9-CM codes and ATC codes used in this study. Table S2: The number of patients in each category of antihypertensive medications and combination therapies. Table S3: Hazard ratios for Parkinson's disease associated with calcium channel blockers, angiotensin converting enzyme inhibitors and angiotensin receptor blockers as compared with beta blockers among different subgroups of patients with hypertension. Table S4: Hazard ratios for Parkinson's disease associated with central and peripheral-acting calcium channel blockers, as compared with beta blockers in patients with hypertension. (DOC) [file pone.0098961.s002.doc]

**Table S1.** ICD-9-CM codes and ATC codes used in this study

| **Comorbidities** | **ICD-9-CM codes** | **Medication** | **ATC codes** |
| --- | --- | --- | --- |
| Diabetes | 250 | Angiotensin receptor blockers | C09CA01, C09CA03, C09CA04, C09CA06, C09CA07, C09CA08, |
| Ischemic heart disease | 410-414 | ACE inhibitors | C09AA |
| Myocardial infarction | 410, 412 | Alpha-blockers | C02CA |
| Angina |  | Beta-blockers | C07A |
| Congestive heart failure | 428 | Calcium channel blockers | C08 |
| Migraine | 346 | COX-2 nonselective NSAIDs | M01A (exclude M01AH, M01AX05) |
| Gout | 274 | COX-2 selective NSAIDs | M01AH |
| Peripheral arterial disease | 440.2, 440.4, 443.81, 443.9 | Anti-platelet agents | B01AC04, B01AC06, N02BA01 |
| Chronic renal failure | 403.01, 403.11, 403.91, 404.02, 404.03, 404.12, 404.13, 404.92, 404.93, 585 ,V45.1, V56.0, V56.8 | Warfarin | B01AA03 |
| Chronic liver disease | 070.2x, 070.3x, V02.61, 070.41, 070.44, 070.51, 070.54, V02.62, 571.0, 571.1, 571.2, 571.3, 571.4, 571.5, 571.6 | Statins | C10AA |
| Chronic obstructive lung disease | 490-496, 500-508 | Nitrate | C01DA |
| Chronic obstructive pulmonary disease | 491, 492, 494, | Anti-diabetic agents | A10B |
| Seizure | 345, 780.3 | Insulin | A10A |
| Rheumatoid arthritis | 710, 714, 720 | Fibrates | C10AB |
| Osteoarthritis | 715 | Diuretics | C03 |
| Osteoporosis | 733.0 | Anti-arrhythmics | C01B |
| Depression | 296.2,296.3,298.0,300.4,309.0,309.1, 293.83,296.90,309.28,296.82, 311 | Estrogen | G03C |
| Anxiety disorder | 300.0-300.3, 300.5-300.9 | Digitalis | C01AA |
| Bipolar disorder | 296.0, 296.1, 296.4- 296.9 | Anti–psychotics | N05A |
| Psychotic disorder | 290.8, 290.9, 780.1, 295, 297-299 | Anti-depressants | N06A |
| Peptic ulcer disease | 531-534 | Anti-epileptics | N03A |
| Thyroid disease | 244.9 | Thyroid therapy | H03 |
| Cancer | 140-208 | Anti-gout preparations | M04A |

**Table S2.** The number of patients in each category of antihypertensive medications and combination therapies.

| Drug category | Patient number | Percentage of total enrolled patients |
| --- | --- | --- |
| ACEI | 8153 | 12.54% |
| ARB | 5298 | 8.15% |
| CCB | 20503 | 31.54% |
| BB | 10522 | 16.19% |
| ACEI+BB | 1622 | 2.50% |
| ACEI+CCB | 3341 | 5.14% |
| ARB+BB | 1594 | 2.45% |
| ARB+CCB | 3336 | 5.13% |
| BB+CCB | 6446 | 9.92% |
| ACEI+BB+CCB | 1033 | 1.59% |
| ARB+BB+CCB | 1406 | 2.16% |
| Others | 100 | 2.69% |

ACEI, angiotensin converting enzyme inhibitors; ARB, angiotensin receptor blockers; CCB, calcium channel blockers; BB, beta-blockers.

**Table S3.** Hazard ratios for Parkinson’s disease associated with calcium channel blockers, angiotensin converting enzyme inhibitors and angiotensin receptor blockers as compared with beta blockers among different subgroups of patients with hypertension

|  | Calcium channel blockers | | ACE inhibitors | | Angiotensin receptor blockers | |
| --- | --- | --- | --- | --- | --- | --- |
|  | Crude HR | Adjusted HR§ | Crude HR | Adjusted HR§ | Crude HR | Adjusted HR§ |
| **Female (N=35,059)** |  |  |  |  |  |  |
| Main analysis | 0.78 (0.56–1.09) | 0.66 (0.47–0.93) | 0.73 (0.53–1.00) | 0.76 (0.56–1.05) | 0.87 (0.64–1.19) | 0.81 (0.59–1.11) |
| Dose effect^¶^ |  |  |  |  |  |  |
| 1^st^ quartile (lowest) | 0.67 (0.44–1.03) | 0.76 (0.49–1.18) | 1.22 (0.75–1.99) | 1.29 (0.79–2.10) | 1.38 (0.89–2.15) | 1.42 (0.91–2.22) |
| 2^nd^ quartile | 0.67 (0.45–1.01) | 0.79 (0.52–1.18) | 0.78 (0.44–1.38) | 0.85 (0.48–1.52) | 0.68 (0.38–1.21) | 0.73 (0.41–1.30) |
| 3^rd^ quartile | 0.46 (0.29–0.72) | 0.50 (0.32–0.79) | 0.46 (0.23–0.93) | 0.54 (0.29–1.09) | 0.64 (0.37–1.11) | 0.65 (0.37–1.14) |
| 4^th^ quartile (highest) | 0.51 (0.33–0.78) | 0.51 (0.33–0.77) | 0.46 (0.25–0.84) | 0.54 (0.29–0.99) | 0.38 (0.19–0.73) | 0.37 (0.19–0.72) |
| **Male (N=29,942)** |  |  |  |  |  |  |
| Main analysis | 1.01 (0.71–1.45) | 0.84 (0.58–1.20) | 0.91 (0.66–1.27) | 0.88 (0.63–1.23) | 1.01 (0.73–1.40) | 0.95 (0.69–1.32) |
| Dose effect^¶^ |  |  |  |  |  |  |
| 1^st^ quartile (lowest) | 0.83 (0.54–1.28) | 0.82 (0.53–1.26) | 1.04 (0.59–1.82) | 0.93 (0.53–1.64) | 0.80 (0.42–1.50) | 0.77 (0.41–1.44) |
| 2^nd^ quartile | 1.07 (0.74–1.54) | 1.06 (0.73–1.54) | 0.69 (0.36–1.30) | 0.69 (0.37–1.30) | 1.05 (0.63–1.74) | 1.04 (0.62–1.73) |
| 3^rd^ quartile | 0.59 (0.37–0.94) | 0.57 (0.36–0.90) | 1.09 (0.67–1.77) | 1.09 (0.67–1.78) | 0.72 (0.41–1.26) | 0.74 (0.42–1.30) |
| 4^th^ quartile (highest) | 0.86 (0.59–1.25) | 0.73 (0.51–1.67) | 0.49 (0.26–0.92) | 0.49 (0.26–0.93) | 0.87 (0.54–1.41) | 0.83 (0.51–1.35) |
| **Age≧65 years (N=33, 548)** |  |  |  |  |  |  |
| Main analysis | 0.74 (0.56–0.97) | 0.74 (0.56–0.97) | 0.75 (0.59–0.97) | 0.83 (0.64–1.06) | 0.90 (0.71–1.15) | 0.92 (0.72–1.17) |
| Dose effect^¶^ |  |  |  |  |  |  |
| 1^st^ quartile (lowest) | 0.67 (0.47–0.95) | 0.76 (0.54–1.08) | 0.91 (0.59–1.41) | 1.01 (0.65–1.56) | 1.07 (0.73–1.58) | 1.19 (0.81–1.76) |
| 2^nd^ quartile | 0.80 (0.59–1.08) | 0.93 (0.69–1.26) | 0.57 (0.34–0.95) | 0.63 (0.38–1.06) | 0.90 (0.60–1.34) | 0.94 (0.63–1.41) |
| 3^rd^ quartile | 0.51 (0.37–0.72) | 0.57 (0.41–0.80) | 0.85 (0.57–1.28) | 0.97 (0.64–1.45) | 0.70 (0.46–1.06) | 0.73 (0.48–1.11) |
| 4^th^ quartile (highest) | 0.61 (0.45–0.82) | 0.65 (0.48–0.87) | 0.56 (0.36–0.86) | 0.64 (0.41–0.98) | 0.54 (0.35–0.83) | 0.57 (0.37–0.87) |
| **Age < 65 years (N=31, 453)** |  |  |  |  |  |  |
| Main analysis | 0.71 (0.41–1.23) | 0.69 (0.40–1.21) | 0.70 (0.41–1.22) | 0.72 (0.41–1.26) | 0.63 (0.36–1.12) | 0.63 (0.35–1.12) |
| Dose effect^¶^ |  |  |  |  |  |  |
| 1^st^ quartile (lowest) | 0.56 (0.25–1.29) | 0.58 (0.25–1.33) | 1.44 (0.67–3.09) | 1.42 (0.66–3.06) | 0.80 (0.29–2.16) | 0.83 (0.31–2.26) |
| 2^nd^ quartile | 1.06 (0.60–1.89) | 1.12 (0.63–2.00) | 0.92 (0.38–2.27) | 0.96 (0.39–2.37) | 0.48 (0.15–1.50) | 0.49 (0.16–1.56) |
| 3^rd^ quartile | 0.23 (0.07–0.72) | 0.23 (0.07–0.74) | 0.63 (0.23–1.70) | 0.64 (0.24–1.75) | 0.42 (0.13–1.33) | 0.43 (0.14–1.37) |
| 4^th^ quartile (highest) | 0.50 (0.23–1.08) | 0.51 (0.24–1.10) | NA | NA | 0.65 (0.26–1.58) | 0.64 (0.26–1.59) |

§Stratified by baseline disease risk score deciles and adjusted for time varying co-morbidities and medications use (statins, anti-diabetes, anti-platelets, non-steroidal anti-inflammatory drugs, anti-psychotics, ischemic heart disease, heart failure, chronic kidney disease, and chronic obstructive pulmonary disease)

^¶^ Calculated as quartiles of cumulative defined daily dose (DDD) of the drug class category

ACE, angiotensin converting enzyme; HR, hazard ratio.

**Table S4.** Hazard ratios for Parkinson’s disease associated with central and peripheral-acting calcium channel blockers, as compared with beta blockers in patients with hypertension.

|  | | Central-acting CCBs^b^ | | | Peripheral-acting CCBs^c^ | |
| --- | --- | --- | --- | --- | --- | --- |
|  | Crude HR | | Adjusted HR ^a^ | Crude HR | | Adjusted HR ^a^ |
| Main analysis | 0.74(0.59-0.93) | | 0.69(0.55-0.87) | 0.95(0.77-1.19) | | 0.84(0.67-1.04) |
| Dose effect ^e^ |  | |  |  | |  |
| 1^st^ (Lowest quartile) | 0.63(0.41-0.96) | | 0.63(0.42-0.96) | 0.87(0.61-1.23) | | 0.84(0.57-1.19) |
| 2^nd^ | 0.83(0.58-1.19) | | 0.89(0.62-1.28) | 0.94(0.68-1.31) | | 0.96(0.69-1.33) |
| 3^rd^ | 0.70(0.48-1.02) | | 0.73(0.50-1.06) | 0.58(0.40-0.84) | | 0.59(0.41-0.86) |
| 4^th^ (Highest quartile) | 0.57(0.39-0.84) | | 0.54(0.37-0.80) | 0.67(0.49-0.92) | | 0.63(0.46-0.86) |

CCBs calcium channel blockers; HR, hazard ratio.

^a^ Stratified by baseline disease risk score deciles and adjusted for time varying co-morbidities and medications use (statins, anti-diabetes, anti-platelets, non-steroidal anti-inflammatory drugs, anti-psychotics, ischemic heart disease, heart failure, chronic kidney disease, and chronic obstructive pulmonary disease)

^b^ felodipine, nifedipine , lercanidipine , nitrendipine and lacidipine

^c^ verapamil, diltiazem and amlodipine
